# Cardiac and Pulmonary Vascular Remodeling in Endurance Open Water Swimmers Assessed by Cardiac Magnetic Resonance: Impact of Sex and Sport Discipline

**DOI:** 10.3389/fcvm.2021.719113

**Published:** 2021-08-20

**Authors:** Vanessa Martínez, María Sanz-de la Garza, Blanca Domenech-Ximenos, César Fernández, Ana García-Alvarez, Susanna Prat-González, Carles Yanguas, Marta Sitges

**Affiliations:** ^1^Department of Cardiology, Fundació Althaia, Xarxa Assistencial Universitaria de Manresa, Manresa, Spain; ^2^Hospital Clínic, Cardiovascular Institute, IDIBAPS, University of Barcelona, Barcelona, Spain; ^3^Department of Radiology, Hospital Clínic, University of Barcelona, Barcelona, Spain; ^4^Department of Radiology, Fundació Althaia, Xarxa Assistencial Universitaria de Manresa, Barcelona, Spain

**Keywords:** athletes' heart, endurance swimmers, pulmonary circulation, right ventricle, cardiac remodeling

## Abstract

**Background:** The cardiac response to endurance exercise has been studied previously, and recent reports have described the extension of this remodeling to the pulmonary vasculature. However, these reports have focused primarily on land-based sports and few data are available on exercise-induced cardio-pulmonary adaptation in swimming. Nor has the impact of sex on this exercise-induced cardio-pulmonary remodeling been studied in depth. The main aim of our study was to evaluate cardiac and pulmonary circulation remodeling in endurance swimmers. Among the secondary objectives, we evaluate the impact of sex and endurance sport discipline on this cardio-pulmonary remodeling promoted by exercise training.

**Methods:**Resting cardiovascular magnetic resonance imaging was performed in 30 healthy well-trained endurance swimmers (83.3% male) and in 19 terrestrial endurance athletes (79% male) to assess biventricular dimensions and function. Pulmonary artery dimensions and flow as well as estimates of pulmonary vascular resistance (PVR) were also evaluated.

**Results:**In relation to the reference parameters for the non-athletic population, male endurance swimmers had larger biventricular and pulmonary artery size (7.4 ± 1.0 vs. 5.9 ± 1.1 cm^2^, *p* < 0.001) with lower biventricular ejection fraction (EF) (left ventricular (LV) EF: 58 ± 4.4 vs. 67 ± 4.5 %, *p* < 0.001; right ventricular (RV) EF: 60 ± 4 vs. 66 ± 6 %, *p* < 0.001), LV end-diastolic volume (EDV): 106 ± 11 vs. 80 ± 9 ml/m^2^, *p* < 0.001; RV EDV: 101 ± 14 vs. 83 ± 12 ml/m^2^, *p* < 0.001). Significantly larger LV volume and lower LV EF were also observed in female swimmers (LV EF: 60 ± 5.3 vs. 67 ± 4.6 %, *p* = 0.003; LV EDV: 90 ± 17.6 vs. 75± 8.7 ml/m^2^, *p* = 0.002). Compared to terrestrial endurance athletes, swimmers showed increased LV indexed mass (75.0 ± 12.8 vs. 61.5 ± 10.0 g/m^2^, *p* < 0.001). The two groups of endurance athletes had similar pulmonary artery remodeling.

**Conclusions:** Cardiac response to endurance swimming training implies an adaptation of both ventricular and pulmonary vasculature, as in the case of terrestrial endurance athletes. Cardio-pulmonary remodeling seems to be less extensive in female than in male swimmers.

## Introduction

Long-lasting endurance exercise training induces structural and functional remodeling of all four cardiac chambers (1, 2), affecting mainly the right ventricle (RV) rather than the left ventricle (LV) (3, 4). It has also been suggested that exercise-induced remodeling not only involves cardiac chambers but also pulmonary circulation, increasing pulmonary artery dimensions, reducing pulmonary artery flow velocity, and finally increasing the estimated pulmonary vascular resistance (PVR) (5).

Different patterns of cardiac adaptation to endurance exercise have been observed between athletes of different disciplines (6–8). In this regard, previous studies have compared upper- and lower-body endurance training and have shown specific differences in ventricular and atrial remodeling (9, 10). Nevertheless, most of the evidence comparing endurance disciplines involving different muscle groups comes from echocardiographic studies and has focused on LV. Cardiac magnetic resonance (CMR) is considered the gold standard imaging modality for the quantification of biventricular morphology and function (11); additionally, it is also regarded as a non-invasive alternative for evaluating pulmonary circulation hemodynamics and for estimating PVR (12). To our knowledge, most of the CMR studies carried out in male athletes have been performed on subjects practicing terrestrial sports (13–15), and little is known about the hemodynamic effects of swimming on cardiac performance and pulmonary circulation. The effects of swimming on cardiac and pulmonary circulation remodeling may differ from those observed in other sports, given the predominant use of the upper-body and also, presumably, the differential impact of gravitational forces due to the horizontal position and the intermittent respiratory pattern while exercising (16–18).

Consequently, this study had three aims: (1) by means of CMR imaging, to analyze cardiac and pulmonary circulation remodeling in a series of endurance open-water swimmers; (2) to evaluate the impact of the endurance sport discipline, by comparing the remodeling observed in swimmers to that observed in a cohort of terrestrial endurance athletes; and (3) to analyze the impact of sex on this exercise-induced cardio-pulmonary remodeling.

## Methods

### Study Population

Thirty non-elite endurance-trained swimmers taking part in the open water swimming race “Oceanman Palamós 9.5 Km” in Catalonia, Spain, and currently engaged in endurance open-water swimming competitions of distances between 3 and 10 km, volunteered to participate in the study. All athletes were trained primarily using the front stroke technique.

Additionally, data on 19 healthy age and sex-matched non-elite endurance athletes trained in terrestrial sports (cycling and running) were obtained retrospectively from a pre-analyzed CMR data base managed by our working group.

A cardiovascular evaluation including a comprehensive personal and family history, resting 12-lead electrocardiogram, treadmill stress test and transthoracic echocardiography at rest was performed prior to the study in order to rule out cardiovascular disease.

All participants filled out a questionnaire providing details of their training history. Exclusion criteria included any history of cardiopulmonary disease and standard contraindications for CMR imaging. To assess training load, a standardized form based on the IPAQ questionnaire (19) was used and the data collected were presented as MET/min/week. The competitive experience was also recorded.

The study protocol was approved by the ethics committee of our institution and complied with the Declaration of Helsinki.

### Cardiovascular Magnetic Resonance Protocol

The CMR studies in the swimming group were carried out by an experienced cardiologist on a 1.5 Tesla scanner (MAGNETOM Essenza, Siemens Healthcare) using standard steady-state, free precession ECG-gated breath-hold cines in three long-axis planes and sequential short axis slices from the atrioventricular ring to the apex. Additional cross-sectional cine images of main pulmonary artery and ascending aorta at level of right pulmonary artery were acquired. For flow measurements, standard breath-hold velocity-encoded phase-contrast MR sequences were used with slices positioned perpendicular to the long axis of the ascending aorta where the flow is parallel to the long-axis of the body, and perpendicular to the long axis of the main pulmonary artery. The CMR studies in the group of terrestrial athletes were conducted previously using the same acquisition protocol, and the images were also analyzed beforehand by the same cardiologist.

**Figure 1:**
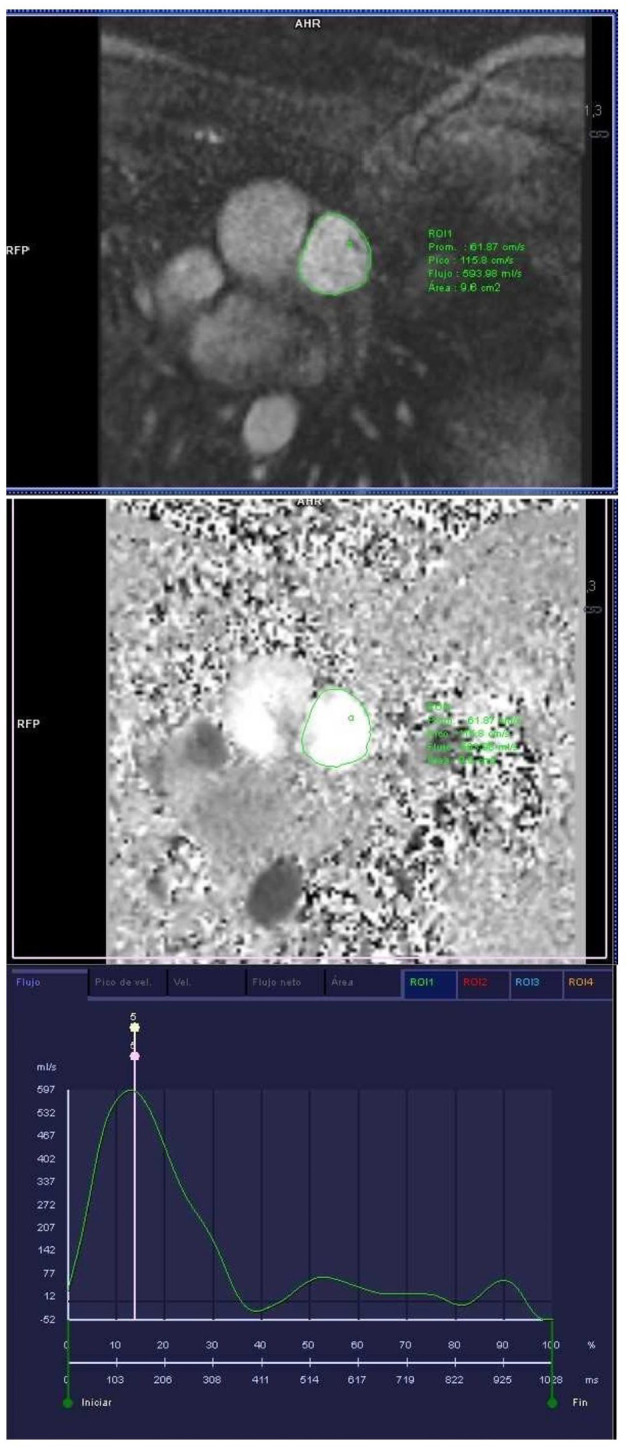
Phase contrast sequence at the level of the main pulmonary artery for the study of pulmonary artery velocities and volumes and subsequent calculation of PVR.

Quantitative image data analysis was performed on a separate workstation using software from the vendor (*syngo.via, Siemens Healthcare, Erlangen, Germany*). Global LV and RV function were assessed by manually contouring the endocardial borders of end-diastolic and end-systolic short-axis images. For the computation of LV mass, the epicardial borders were additionally contoured at end diastole. LV and RV volumes and EF and left ventricular mass were automatically calculated by the software. Measurements were indexed for body surface area. For the assessment of ascending aorta and pulmonary artery flow, the cross-sectional area of these vessels was defined and manually corrected, and flow parameters were then automatically provided by the software (Figure 1). The following parameters were obtained: peak velocity, average velocity during the complete cardiac cycle, minimum and maximum areas, and pulmonary artery net forward volume. Pulmonary artery pulsatility was calculated as: (maximal pulmonary artery area-minimal pulmonary artery area)/minimal pulmonary artery area x 100. PVR was estimated by a formula that was developed from a cohort of patients with diagnosis or suspicion of pulmonary hypertension and validated in an independent cohort of patients (12). Late gadolinium enhancement sequences were not performed because of the need for contrast in healthy volunteer athletes.

Inter-observer intra-class correlations for CMR measurements were performed in 10 endurance swimmers and presented the following results: 0.970 for LV EF, 0.872 for indexed LV EDV, 0.976 for RVEF, 0.916 for indexed RV EDV, 0.960 for pulmonary flow, 0.986 for mean pulmonary flow velocity, 0.983 for indexed maximal pulmonary area and 0.972 for PVR. Regarding terrestrial athletes, concordance was high between the two observers for ejection fraction, EDV and pulmonary artery and average flow velocities (intraclass correlation coefficient-absolute agreement was >0.85 for all five parameters) (5).

### Statistical Analysis

Statistical analysis was carried out using the SPSS Software version 22.0 *(SPSS Inc., Chicago, Illinois, USA)*. All continuous variables were analyzed for normality of distribution using the Kolmogorov-Smirnov test. Subsequently, the Student's *t* test was used to compare baseline data. If normality was not confirmed, the Mann–Whitney-*U* test was applied. Bivariate correlational analysis was used for RV EF, indexed RV EDV, and RVP to determine any possible relationship. The intraclass correlation coefficient (ICC) for the two-way mixed effect models and for absolute agreement ICC (A,2) was calculated to assess the inter-rater reliability of the CMR parameters. The alpha value was set at 0.003 to account for Bonferroni correction.

## Results

Clinical and functional characteristics of both groups and according to sex are shown in Table 1. Irrespective of sex, endurance swimmers and terrestrial athletes had similar training loads and there were no significant differences in mean age. The proportion of women was also similar between the groups (16.7% in swimmers vs. 21.1% in control group, *p* = 0.706). No significant differences were found between male and female swimmers, except for BSA, which was higher in men.

**Table 1:** Clinical and functional characteristics.

**Parameters**	**Swimmers** **(men) (** ***n*** **= 25)**	**Terrestrial endurance athletes (men)** **(** ***n*** **= 15)**	***p*** **-value**	**Swimmers (women)** **(** ***n*** **= 5)**	**Terrestrial endurance athletes (women)** **(** ***n*** **= 4)**	***p*** **-value**
Age (years)	42.2 ± 7.0	39.0 ± 5.4	0.130	37 ± 7.5	35.6 ± 7.6	0.813
BSA (m^2^)	1.9 ± 0.1	1.9 ± 0.2	0.420	1.7 ± 0.1^*^	1.7 ± 0.1	0.909
Training load (MET's/min/week)	5,769 ± 1,584	6,624 ± 1,944	0.138	5,688 ± 864	5,328 ± 720	0.527
Training load (years)	10.6 ± 6.1	13.3 ± 4.2	0.110	10.2 ± 4.4	8.3 ± 1.1	0.154

*BSA, body surface area*.

* *, p < 0.05 for the sex-comparison in swimmers*.

CMR results in endurance swimmers are summarized in Table 2. Compared to the reference parameters reported for the general population (20, 21), male athletes had lower biventricular EF, with larger LV and RV volumes as well as pulmonary artery size (22), and a trend toward higher estimated PVR (5).

**Table 2:** Cardiac magnetic resonance parameters in endurance swimmers according to sex.

**Parameters**	**Swimmers (men) (** ***n*** **= 25)**	Reference value^*^	***p*** **-value**	**Swimmers (women)** **(** ***n*** **= 5)**	Reference value^*^	***p*** **-value**
LV EF (%)	58 ± 4.4	67 ± 4.5	**<0.001**	60 ± 5.3^†^	67 ± 4.6	**0.003**
LV EDV (ml/m^2^)	106 ± 11	80 ± 9	**<0.001**	90 ± 17.6	75 ± 8.7	**0.002**
LV SV (ml/ m^2^)	62 ± 8	53 ± 6	**<0.001**	55± 15.5	50 ± 6.2	0.163
LV Mass (g/m^2^)	78 ± 10.8	74 ± 8.5	0.06	59 ± 10.2^†^	63 ± 7.5	0.257
RV EF	60 ± 4	66 ± 6	**<0.001**	66 ± 4^†^	66 ± 6	0.971
RV EDV (ml/m^2^)	101 ± 14	83 ± 12	**<0.001**	75 ± 15^†^	73 ± 9	0.619
RV SV (ml/m^2^)	60 ± 8	54 ± 8	**0.001**	50 ± 13	48 ± 6	0.498
Pulmonary artery area (cm^2^)	7.4 ± 1.0	3.7 ± 0.7	**<0.001**	5.7 ± 0.5	3.9 ± 0.6	0.128
PVR (Wood Units)	2.3 ± 0.8	1.8 ± 1.2^‡^	0.068	1.3 ± 0.4^†^	1.6 ± 1.1^‡^	0.554

*Values expressed as mean ± SD*.

*LV, left ventricle; EF, ejection fraction; EDV, end-diastolic volume; SV, stroke volume; RV, right ventricle; PVR, pulmonary vascular resistance*.

* *: reference value for the non-athletic population (20, 21)*.

† *, p < 0.05 for the comparison between male and females*.

‡ *, reference values obtained from Domenech-Ximenos et al. (5)*.

*Values in bold indicate significant differences between groups*.

Similar to the men, female swimmers showed significantly lower values of LV EF with larger LV volumes. Conversely, RV dimensions and EF, as well as pulmonary artery size and estimated PVR, were within reference values for the healthy non-athletic population.

Compared to male swimmers, females had higher biventricular EF, smaller LV mass and RV end-diastolic volume (RV EDV) and lower estimated PVR.

### Comparison Between Swimmers and Terrestrial Endurance Athletes

The comparison of CMR parameters between swimmers and controls is shown in Table 3. Swimmers had larger LV mass than terrestrial athletes. Despite a trend toward lesser RV remodeling in swimmers (with smaller RV volumes) than in controls, no significant differences were observed in biventricular dimensions or function. Nor were significant differences found between sport disciplines with regard to pulmonary artery remodeling.

**Table 3:** Cardiac magnetic resonance parameters comparation between swimmers and terrestrial endurance athletes.

**Parameters**	**Swimmers** **(** ***n*** **= 30)**	**Terrestrial endurance athletes** **(** ***n*** **= 19)**	***p*** **-value**
LV EF (%)	56.0 ± 4.6	56.7 ± 5.4	0.918
LV EDV (ml/m^2^)	98.0 ± 12.6	102.8 ± 13.8	0.219
LV SV (ml/ m^2^)	55.0 ± 8.3	58.2 ± 8.1	0.182
LV Mass (g/m^2^)	75.0 ± 12.8	61.5 ± 10.0	**<0.001**
RV EF	56.5 ± 6.6	53.5± 6.8	0.242
RV EDV (ml/m^2^)	96.8 ± 15.4	103.7 ± 18.2	0.164
RV SV (ml/m^2^)	54.3 ± 7.8	55.6 ± 10.9	0.640
Pulmonary artery pulsatility (%)	46.9 ± 12.0	54.9 ± 25.0	0.137
Pulmonary artery area (cm^2^/m^2^)	3.8 ± 0.4	4.0 ± 0.6	0.131
Mean pulmonary velocity (cm/s)	16.0 ± 2.8	16.3 ± 2.4	0.646
PVR (Wood Units)	2.2 ± 0.9	2.2 ± 0.8	0.800

*Values expressed as mean ± SD*.

*LV, left ventricle; EF, ejection fraction; EDV, end-diastolic volume; SV, stroke volume*.

*RV, right ventricle; PVR, pulmonary vascular resistance*.

*Values in bold indicate significant differences between groups*.

**Figure 2:**
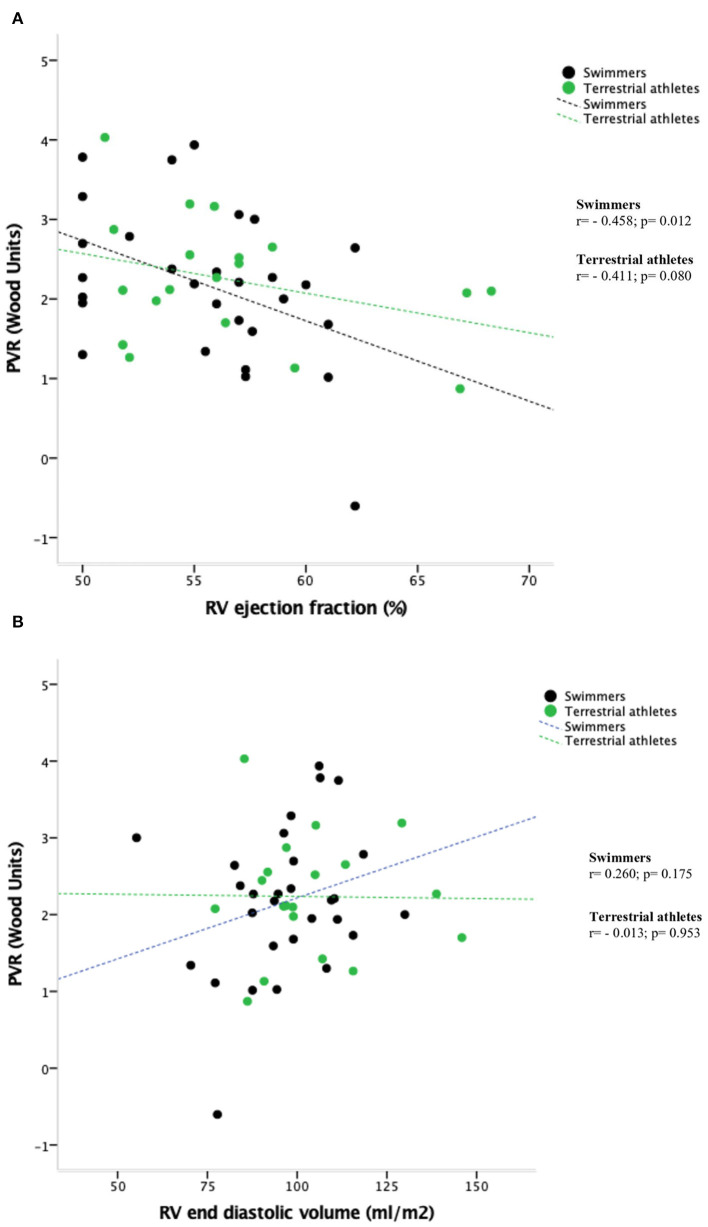
**(A)** Relationship between RV ejection fraction and cardiac-magnetic resonance-estimated pulmonary resistance (PVR) values. **(B)** Relationship between RV end diastolic volume and cardiac-magnetic resonance-estimated pulmonary resistance (PVR) values.

Figures 2A,B show the relationship between RV EF and RV EDV respectively with PVR. Swimmers with higher PVR presented more RV remodeling, as indicated by lower RV EF and RV EDV. No significant correlations were observed in terrestrial athletes.

## Discussion

The current study presents a comprehensive CMR evaluation of the impact of endurance swimming on ventricular and pulmonary circulation structure and function. To our knowledge, the effects of endurance swimming training on cardiac and pulmonary vasculature remodeling have not been previously evaluated in CMR studies.

The key findings of our study were: (1) endurance swimming promoted a significant remodeling of both ventricles, characterized by greater biventricular volumes and lower biventricular function as well as significant remodeling of the pulmonary vasculature (PV) with greater pulmonary artery size and increased PVR; (2) sex has a strong impact on the RV and PV remodeling induced by endurance swimming, with female athletes exhibiting smaller RV cavities, higher RV function and lower PVR; (3) endurance sport discipline did not influence the PV remodeling promoted by long-lasting exercise training, as similar pulmonary artery size, and equivalent estimated PVR were observed in swimmers and terrestrial endurance athletes. However, a trend toward lower exercise-induced RV remodeling was shown in swimmers.

Previous studies have evaluated cardiac adaptation to endurance exercise using CMR imaging, showing enlarged LV and RV dimensions with no differences in biventricular EF between endurance athletes and non-athletic populations (11, 23, 24). Nevertheless, most of these studies were performed in athletes practicing terrestrial sports (running and cycling) or triathletes. As expected, our results showed that male endurance swimmers presented significantly larger biventricular volumes than the general population.

We have recently reported that the response to endurance chronic training involves not only the four cardiac chambers but also the pulmonary circulation, since endurance athletes showed greater pulmonary artery dimensions with reduced pulmonary artery flow velocity and a higher estimated PVR than non-athletes (5). In the present study, endurance swimming was also shown to induce pulmonary artery remodeling at least in male swimmers who presented a larger pulmonary artery size and a trend toward higher estimated PVR values than the general population. The increase in pulmonary artery pressure (PAP) during exertion has been established as a potential mechanism for the acute RV impairment observed after bouts of intense exercise (25). If these increases in PAP occur repeatedly over time, they may induce a chronic remodeling of the pulmonary circulation and the right heart. Related to this, Figure 1A shows that individuals with greater PAP remodeling have lower RV function. Our results highlight the importance of including CMR evaluation of pulmonary circulation in endurance athletes in order to assess another aspect of remodeling induced by endurance training: the potentially excessive remodeling of the pulmonary circulation, resulting also in excessive RV remodeling.

However, the cardiopulmonary adaptation differed slightly in female and male swimmers. In females, with respect to the reference population, only enlarged LV and reduced LV EF were observed. Compared to male swimmers, women had smaller LV and RV volumes, higher LV and RV EF, and lower pulmonary artery size and estimated PVR. Thus, the results of the present study show that male swimmers present more extensive biventricular and pulmonary circulation remodeling than their female counterparts. The reason for this difference is not entirely clear, but the finding is in agreement with previous studies performed in idiopathic pulmonary arterial hypertension patients, which have demonstrated a poorer adaptation of RV to increased overload in male subjects (26, 27). This disease is more frequent in women; however, once affected, women present a better prognosis than men. This “estrogen paradox” has been associated with a better hemodynamic profile, more distensible pulmonary circulation and better RV performance (28). Sex hormones seem to play an important role explaining this sex bias, although experimental studies in animal models and clinical observations have yielded conflicting results (29–33).

Using CMR parameters established for the general population as a reference may have induced errors in the athletes' heart evaluations, given that exercise-induced increases in ventricular dimensions may overlap with those observed in patients with pathological conditions, such as dilated cardiomyopathy and RV arrhythmogenic cardiomyopathy. In this connection, a recent meta-analysis defined the normal limits of biventricular size and function in competitive male athletes, which represent a useful tool for athletes' heart evaluations (34). Our results showed ventricular dimensions and function within the reference range reported for competitive athletes. However, we observed a lower remodeling of the RV in swimmers, with RV volumes below, and RV EF above, these limits of reference (34). Additionally, female swimmers showed lower LV remodeling, with LV volumes and LV indexed mass below the reference range.

It has been established that functional and structural cardiac remodeling induced by exercise is influenced by the specific sport discipline; cardiac response to endurance training differs according to the muscle group predominantly involved during exercise (upper- vs. lower-body) (9, 18). The hemodynamic response to sport training may be an important determinant of this ventricular adaptation (35). A recent echocardiographic study comparing cardiac remodeling between endurance swimmers and a cohort of long-distance runners found that swimmers presented lesser RV remodeling (8). So the hemodynamic impact of swimming on cardiac remodeling may differ with regard to other sports, given that swimmers predominantly use the upper-body muscle groups. Additionally, swimming is performed in water, and in a horizontal position, which implies a different impact of gravitational forces on the RV pre-load (16–18). Finally, during swimming, the breath is held for prolonged periods, leading to intermittent hypoxia which in turn induces alveolar hyperplasia; similarly, ventilation is restricted under water and the respiratory muscles have to work under greater load. It has been observed that swimmers have larger lung volume and better pulmonary function than other athletes, with higher values of vital capacity, forced vital capacity (FVC), forced expiratory volume for 1 s (FEV1) and FEV1/FVC), unrelated to anthropometric features or to training history (36, 37). Considering all these aspects, the pulmonary circulatory response in swimmers may differ from that found in other athletes, and this may account for the different right ventricular adaptation to endurance training. In the present study, we only observed a trend toward less extensive RV remodeling (with smaller RV dimensions and function) compared to the control group probably due to the small sample size in both groups and the resulting low statistical power.

Finally, the beneficial effect of exercise in terms of cardiovascular health is well established, and greater benefits are obtained with moderate exercise. The athlete's heart is a result of a series of adaptive mechanisms of the heart to cope with the overload involved in intense long-lasting athletic training. However, the amount of this remodeling can make it difficult to distinguish between physiological and pathological cardiac changes. Additionally, emerging data suggest the existence of a syndrome of exercise-induced arrhythmogenic RV cardiomyopathy (38) which may be related to chronic intense-training in genetically predisposed individuals (39). Thus, distinction between physiological and pathological cardiac remodeling is essential in order to detect individuals who are potentially susceptible to present adverse cardiac events. In this context, previous authors have underlined the importance of the use of advanced echocardiographic methods such as the tissue Doppler and two-dimensional strain to help cardiologists in their study of the diagnostic overlaps in sports cardiology, also known as “gray zones” (40). Taking into account that CMR is considered the gold standard imaging modality for the quantification of biventricular morphology and function, it should be considered a complementary tool in the study of athletes included in these “gray zones”.

## Limitations

The small sample size of this exploratory observational study limits the statistical power and generalizability of the results. Given the low number of women in both groups of endurance athletes, the conclusions drawn from the sex comparison should be considered exploratory. Further studies with larger athlete populations including women are needed to better understand the influence of sex on cardiac remodeling. PVR values were not obtained invasively with right heart catheterization, and the method used to estimate PVR has not been validated in healthy population. However, a previous study by our group (5) found a high concordance in healthy athletes between PVR data obtained by CMR model and direct and indirect echocardiographic estimates of pulmonary pressure at rest and after exercise (5).

As peak oxygen consumption (VO2) was not measured, we cannot rule out a possible effect of VO2 on cardiac remodeling and, therefore, on the results obtained.

We did not perform delayed gadolinium enhancement sequences, thus limiting the analysis of fibrosis induced by chronic endurance swimming.

## Conclusions

Endurance chronic swimming training induces biventricular and pulmonary vascular adaptation which is more pronounced in males. As compared to other endurance athletes, swimmers seem to present a less extensive remodeling of the RV. These findings may be due to a differential hemodynamic impact of swimming on the pulmonary circulation and the right heart.

## Data Availability Statement

The raw data supporting the conclusions of this article will be made available by the authors, without undue reservation.

## Ethics Statement

The studies involving human participants were reviewed and approved by Fundació Catalana Hospitals, approval number CEIC 15/28. The patients/participants provided their written informed consent to participate in this study.

## Author Contributions

VM conceived and designed the study, analyzed the data, and interpreted the results. VM, CF, and CY performed the cardiac magnetic resonance studies. MSG, BD-X, AG-A, SP-G, and MS contributed to the interpretation of the results. VM wrote the manuscript and MS supervised the study. All authors contributed to manuscript revision, read and approved the final manuscript.

## Conflict of Interest

The authors declare that the research was conducted in the absence of any commercial or financial relationships that could be construed as a potential conflict of interest.

## Publisher's Note

All claims expressed in this article are solely those of the authors and do not necessarily represent those of their affiliated organizations, or those of the publisher, the editors and the reviewers. Any product that may be evaluated in this article, or claim that may be made by its manufacturer, is not guaranteed or endorsed by the publisher.
